# Are rare heterozygous *SYNJ1* variants associated with Parkinson’s disease?

**DOI:** 10.1101/2024.05.29.24307986

**Published:** 2024-06-01

**Authors:** Konstantin Senkevich, Sitki Cem Parlar, Cloe Chantereault, Eric Yu, Jamil Ahmad, Jennifer A. Ruskey, Farnaz Asayesh, Dan Spiegelman, Cheryl Waters, Oury Monchi, Yves Dauvilliers, Nicolas Dupré, Irina Miliukhina, Alla Timofeeva, Anton Emelyanov, Sofya Pchelina, Lior Greenbaum, Sharon Hassin-Baer, Roy N. Alcalay, Ziv Gan-Or

**Affiliations:** 1. The Neuro (Montreal Neurological Institute-Hospital), McGill University, Montreal, Quebec, Canada; 2. Department of Neurology and neurosurgery, McGill University, Montréal, QC, Canada, Canada; 3. Department of Human Genetics, McGill University, Montréal, QC, Canada; 4. Department of Neurology, College of Physicians and Surgeons, Columbia University Medical Center, NY, USA.; 5. Département de radiologie, radio-oncologie et médecine nucléaire, Université de Montréal, Montréal, QC, Canada.; 6. Centre de recherche de l’Institut universitaire de gériatrie de Montréal, Montréal, QC, Canada; 7. National Reference Center for Narcolepsy, Sleep Unit, Department of Neurology, Guide-Chauliac Hospital, CHU Montpellier, University of Montpellier, Montpellier, France.; 8. Neuroscience axis, CHU de Québec-Université Laval, Québec, QC, Canada.; 9. Department of Medicine, Faculty of Medicine, Université Laval, Quebec City, QC, Canada; 10. Institute of the Human Brain of RAS, St. Petersburg, Russia.; 11. First Pavlov State Medical University of St. Petersburg, Saint-Petersburg, Russia; 12. Faculty of Medicine, Tel Aviv University, Tel Aviv, Israel; 13. The Danek Gertner Institute of Human Genetics, Sheba Medical Center, Tel Hashomer, Israel; 14. The Joseph Sagol Neuroscience Center, Sheba Medical Center, Tel Hashomer, Israel; 15. The Movement Disorders Institute, Department of Neurology, Sheba Medical Center, Tel Hashomer, Israel; 16. Division of Movement Disorders, Tel Aviv Sourasky Medical Center, Tel Aviv, Israel.

**Keywords:** Parkinson’s disease, *SYNJ1*, Early-onset Parkinson’s disease, genetics, rare variants

## Abstract

Previous studies have suggested that rare biallelic *SYNJ1* mutations may cause autosomal recessive parkinsonism and Parkinson’s disease (PD). Our study explored the impact of rare *SYNJ1* variants in non-familial settings, including 8,165 PD cases, 818 early-onset PD (EOPD, <50 years) and 70,363 controls. Burden meta-analysis using optimized sequence Kernel association test (SKAT-O) revealed an association between rare nonsynonymous variants in the Sac1 SYNJ1 domain and PD (P_fdr_=0.040). Additionally, a meta-analysis focusing on patients with EOPD demonstrated an association between all rare *SYNJ1* variants and PD (P_fdr_=0.029). Rare *SYNJ1* variants may be associated with sporadic PD, and more specifically with EOPD.

Parkinson’s disease (PD) is a complex condition influenced by multiple genes and environmental factors, with specific single-gene mutations accounting only for 1–2% of cases, excluding *GBA1*^1^. Early onset of PD (EOPD; <50 years) represents about 3–14% of all PD cases depending on population^2^, and has been linked to mutations in several genes (i.e. *PRKN*, *PINK1*, *DJ-1*, *LRRK2, SNCA, GBA1*)^2^. Considering variants in these genes only, the frequency of genetically associated PD in early-onset cases reaches about 16.6%^3^.

Synaptojanin 1, encoded by *SYNJ1*, is a lipid phosphatase abundantly expressed in brain tissues^4^ which plays an important role in synaptic trafficking and in autophagic clearance^5–7^. The protein consists of three functionally distinct domains: Sac1, 5-phosphatase, and proline rich domains (PRD)^8^. Previous studies suggested that biallelic *SYNJ1* mutations cause autosomal recessive, early-onset parkinsonism and EOPD^9–12^. In the current study, we sought to examine the role of rare *SYNJ1* variants in six non-familial cohorts with a total of 8,165 PD cases (including 818 EOPD) and 70,363 controls (detailed in Supplementary Table 1).

The average coverage of the *SYNJ1* gene in the four cohorts sequenced at McGill was >4000X, with 100% of nucleotides covered at >30X (Supplementary Table 2). From these cohorts, 23–75 rare variants were included in the analysis, depending on the cohort (detailed in Supplementary Table 3). In the AMP-PD cohort, 523 rare variants were included in the analysis and 440 rare variants were included in the UKBB cohort (coding and functional variants detailed in Supplementary Table 4).

Burden analysis with SKAT-O suggested a possible association between all rare variants and PD in the AMP-PD cohort (P=1.42E-05; P_fdr_=2.98E-04), with nominal associations that did not survive false discovery rate (fdr) correction in the Columbia cohort (P=0.030; P_fdr_=0.126) and the McGill cohort (P=0.009; P_fdr_=0.095). No association was found in the meta-analysis of all six cohorts after false discovery rate correction (P=0.025; P_fdr_=0.131). We also found a nominal association between non-synonymous variants and PD in the Columbia cohort (P=0.012; P_fdr_=0.084; Table 1).

We then analyzed only EOPD (age at onset < 50 years) and found an association between all rare variants and PD in the AMP-PD cohort (P=3.48E-05; P_fdr_=7.31E-04) and nominal association that did not survive FDR correction in the McGill cohort (P=0.027; P_fdr_=0.189). In the meta-analysis, which included only EOPD patients and controls, all rare variants in *SYNJ1* were associated with PD (P=2.80E-03; P_fdr_=0.029). The association between non-synonymous variants and PD was nominally significant in the Columbia cohort (P=0.044; P_fdr_=0.231) but not in the meta-analysis (Table 1).

To analyze variants within specific functional domains of SYNJ1 (Sac1, 5-phosphatase, PRD), we divided the gene regions into these domains and then repeated SKAT-O analysis. We found an association between the Sac1 domain of SYNJ1 and PD for non-synonymous variants with high CADD scores in the UKBB cohort (P=0.006; P_fdr_=0.082), which did not survive fdr correction. However, this association became significant in the meta-analysis (P=0.002; P_fdr_=0.04; Supplementary Table 5). We observed that this association was primary driven by the p.A195T *SYNJ1* variant in the UKBB cohort (Odds ratio (OR)=4.87; 95% confidence interval (CI) 1.76–13.46, P=0.002, with a minor allele frequency (MAF) in cases of 0.001 and 0.0002 in controls and gnomAD). This variant is classified as a Variant of Uncertain Significance (VUS) in ClinVar and is likely benign according to the ACMG classification but is noted for its high CADD score. This variant was also detected in one patient from the Pavlov and Human Brain cohort and was not reported in any other studied cohorts.

We did not find any homozygous or compound heterozygous carriers of pathogenic variants (previously reported in association with PD^10^, defined as pathogenic or likely pathogenic by ClinVar or variants with CADD score >20) in none of the studied cohorts.

In the current study, we found an association between all rare heterozygous variants in *SYNJ1* and heterozygous variants with high CADD score in the Sac1 domain of SYNJ1, and the risk of PD in some of the analyzed cohorts. These findings suggest that *SYNJ1* could be associated with sporadic PD. Additional studies are required to determine whether this potential association holds in other cohorts. The association we found of rare heterozygous *SYNJ1* variants with EOPD was more convincing, yet here too, additional studies are needed. We did not identify biallelic carriers in our analysis, although private bi-allelic *SYNJ1* variants were previously associated with EOPD and atypical parkinsonism (detailed in^10^),

Multiple genes that are involved in the autophagy-lysosomal pathway are also associated with PD^13^. From the biological point of view, *SYNJ1* has a role in two pathways relevant to PD: synaptic trafficking and autophagic clearance^6,7^. Recent functional studies demonstrated that mutations in *SYNJ1* destabilize dopaminergic neurons potentially due to defective clathrin uncoating, disrupting lipid metabolism and synaptic function^14,15^. However, SYNJ1 overexpression can counteract these effects, highlighting its potential therapeutic potential in PD^14,15^.

Our study has several limitations. In some of our cohorts, we had significant differences in sex and age between PD patients and controls. We adjusted for the effects of these covariates in our analysis to address this limitation. Additionally, in our study we predominantly included participants with European ancestry. Last, we different types of sequencing data and quality control procedures were performed independently for each of the cohorts. Thus, it could potentially create differences in variant enrichment between cohorts. To partially overcome this limitation, we analyzed each cohort separately and conducted meta-analyses of the different cohorts, rather than joint analysis of all cohorts.

To conclude, we found that rare heterozygous *SYNJ1* variants were potentially associated with EOPD and variants in the Sac1 domain are associated with sporadic PD. Larger studies in cohorts of different ethnic backgrounds are needed to replicate our results.

## Methods

### Population

The study population comprised 8,165 PD patients and 70,363 controls from six cohorts, including 818 EOPD (all demographic characteristics detailed in Supplementary Table 1). Four cohorts were collected at McGill university: (1) a cohort of French/French-Canadian from Quebec, Canada and Montpellier, France, (2) a cohort from Columbia University (New York, NY), (3) a cohort from Sheba Medical Center (Israel) and (4) a cohort from Pavlov and Human Brain institutes (Russia).^16^The Columbia cohort comprises patients and controls of varied racial and ethnic origin (European, Ashkenazi [AJ] descent and a minority of Hispanics and blacks). Patients and controls in the Sheba cohort, which was recruited in Israel, are of full AJ ancestry (all four grandparents are AJ). The Pavlov and Human Brain institute cohort was collected from the North-Western region of Russia and mainly with East-European ancestry. Additionally, we performed the analysis in the Accelerating Medicines Partnership – Parkinson Disease (AMP-PD) initiative cohorts (https://amp-pd.org/; detailed in the Acknowledgment) and the UK biobank (UKBB) cohort. We only included participants of European ancestry from both cohorts, and we excluded any first and second-degree relatives from the analysis.

All PD patients were diagnosed by movement disorder specialists according to the UK brain bank criteria^17^ or the MDS clinical diagnostic criteria^18^.

### Standard Protocol Approvals, Registrations, and Patient Consents

All local IRBs approved the protocols and informed consent was obtained from all individual participants before entering the study.

### Targeted next-generation sequencing by molecular inversion probes

The entire coding sequence of the *SYNJ1* gene, including exon-intron boundaries (±50bps) and the 5’ and 3’ untranslated regions (UTRs), was targeted using molecular inversion probes (MIPs) as described earlier^19^. The full protocol is available at https://github.com/gan-orlab/MIP_protocol. The Genome Quebec Innovation Centre’s Illumina NovaSeq 6000 SP PE100 platform was used to sequence the library. Alignment was carried out to hg19 reference genome^20^ with coordinates for *SYNJ1* chr21:34,001,069–34,100,351. Genome Analysis Toolkit (GATK, v3.8) was used for post-alignment quality checking and variant calling^21^. We applied standard quality control procedures as described before^22^. In brief, using the PLINK program version 1.9 and GATK, v3.8, we carried out quality control by eliminating variants and samples with poor quality. SNPs and samples with genotyping rate lower than 90% were excluded. The analyses only included variants with 30x minimal depths of coverage, having a MAF less than 1% and a minimum quality score (GQ) of 30.

### Whole-exome and whole-genome sequencing data

The genetic data in AMP-PD and UKBB were aligned to the human reference genome hg38 and we used the appropriate coordinates to extract the *SYNJ1* data (chr21:32,628,759–32,727,939). Quality control procedures were performed as previously described in detail for AMP-PD^23^ and UKBB^24^ cohorts.

### Statistical Analysis

To analyze rare variants (MAF < 0.01), we applied the optimized sequence Kernel association test (SKAT-O, R package)^25^ in each cohort separately, followed by meta-analysis using the metaSKAT package^26^. We performed separate analyses for all rare variants, non-synonymous variants, and variants with Combined Annotation Dependent Depletion (CADD) scores of ≥ 20. For domain-based analysis, domains boundaries were decided by the widest intervals of each domain based on a combination of estimates from publicly available domain annotation resources. These resources are SuperFamily, Pfam, Smart, Gene3D, PANTHER, Conserved Domains Database, and PROSITE^27–30^. We adjusted for sex, age and ethnicity in all analyses. FDR correction was applied to all p-values.

## Supplementary Material

Supplement 1

## Figures and Tables

**Table 1.** Burden analysis of rare *SYNJ1* variants

		Burden analysis in full cohort (n=8,165 cases and n=70,363 controls)			Burden analysis in early onset (<50) cases only (n=818 cases and n=7,427 controls)		
Cohort	Variants included in analysis	P-value	P_fdr_	N of varaints	P-value	P_fdr_	N of vairants
AMP_PD	All rare	**1.42E-05**	**2.98E-04**	523	**3.48E-05**	**7.31E-04**	471
AMP_PD	Nonsynonymous	0.241	0.633	26	0.273	0.956	23
AMP_PD	CADD	0.248	0.579	9	0.298	0.894	9
Columbia	All rare	0.030	0.126	66	0.574	0.927	40
Columbia	Nonsynonymous	0.012	0.084	25	0.044	0.231	15
Columbia	CADD	0.641	0.841	15	0.375	0.984	7
McGill	All rare	0.009	0.095	62	0.027	0.189	31
McGill	Nonsynonymous	0.693	0.809	24	0.573	1.000	13
McGill	CADD	0.665	0.821	19	0.592	0.888	10
Pavlov and Human brain	All rare	0.448	0.941	75	0.807	0.847	38
Pavlov and Human brain	Nonsynonymous	0.605	0.847	35	0.631	0.779	17
Pavlov and Human brain	CADD	0.578	0.867	37	0.74	0.863	17
Sheba	All rare	0.720	0.796	23	0.608	0.851	16
Sheba	Nonsynonymous	0.733	0.770	8	0.464	0.974	5
Sheba	CADD	0.463	0.884	6	0.405	0.945	6
UKBB	All rare	0.530	0.856	440	0.827	0.825	180
UKBB	Nonsynonymous	0.779	0.779	163	0.464	0.809	24
UKBB	CADD	0.497	0.870	67	0.673	0.831	78
**Meta-analysis**	All rare	**0.025**	0.131		**2.80E-03**	**0.029**	
**Meta-analysis**	Nonsynonymous	0.090	0.315		0.217	0.951	
**Meta-analysis**	CADD	0.120	0.361		0.485	0.953	

UKBB – UK biobank, AMP-PD – Accelerating Medicines Partnership – Parkinson Disease; Combined Annotation Dependent Depletion (CADD) score >20, P_fdr_- p-value with false discovery rate adjustment.

## Data Availability

Data used in the preparation of this article were obtained from the AMP PD Knowledge Platform (https://www.amp-pd.org) and UKBB. Access to these datasets is available for eligible researchers upon request. The code utilized in this study is accessible at https://github.com/gan-orlab/SYNJ1. We provided the variants used for burden analyses in the supplementary tables.
